# Longitudinal magnetic resonance imaging reveals striatal hypertrophy in a rat model of long-term stimulant treatment

**DOI:** 10.1038/tp.2016.158

**Published:** 2016-09-06

**Authors:** D Biezonski, R Shah, A Krivko, J Cha, D N Guilfoyle, J Hrabe, S Gerum, S Xie, Y Duan, R Bansal, B L Leventhal, B S Peterson, C Kellendonk, J Posner

**Affiliations:** 1 Department of Psychiatry, New York State Psychiatric Institute, Columbia University College of Physicians and Surgeons, New York, NY, USA; 2 Center for Biomedical Imaging and Neuromodulation, Nathan S. Kline Institute for Psychiatric Research, Orangeburg, NY, USA; 3 Institute for the Developing Mind, Children's Hospital Los Angeles and the Keck School of Medicine, University of Southern California, Los Angeles, CA, USA; 4 Langley Porter Psychiatric Institute, University of California, San Francisco, San Francisco, CA, USA

## Abstract

Stimulant treatment is highly effective in mitigating symptoms associated with attention-deficit/hyperactivity disorder (ADHD), though the neurobiological underpinnings of this effect have not been established. Studies using anatomical magnetic resonance imaging (MRI) in children with ADHD have suggested that long-term stimulant treatment may improve symptoms of ADHD in part by stimulating striatal hypertrophy. This conclusion is limited, however, as these studies have either used cross-sectional sampling or did not assess the impact of treatment length on their dependent measures. We therefore used longitudinal anatomical MRI in a vehicle-controlled study design to confirm causality regarding stimulant effects on striatal morphology in a rodent model of clinically relevant long-term stimulant treatment. Sprague Dawley rats were orally administered either lisdexamfetamine (LDX, ‘Vyvanse') or vehicle (*N*=12 per group) from postnatal day 25 (PD25, young juvenile) until PD95 (young adult), and imaged one day before and one day after the 70-day course of treatment. Our LDX dosing regimen yielded blood levels of dextroamphetamine comparable to those documented in patients. Longitudinal analysis of striatal volume revealed significant hypertrophy in LDX-treated animals when compared to vehicle-treated controls, with a significant treatment by time point interaction. These findings confirm a causal link between long-term stimulant treatment and striatal hypertrophy, and support utility of longitudinal MRI in rodents as a translational approach for bridging preclinical and clinical research. Having demonstrated comparable morphological effects in both humans and rodents using the same imaging technology, future studies may now use this rodent model to identify the underlying cellular mechanisms and behavioral consequences of stimulant-induced striatal hypertrophy.

## Introduction

Stimulant medication is commonly used to treat symptoms associated with attention-deficit/hyperactivity disorder (ADHD). Acutely, stimulants are thought to confer their therapeutic effects by altering monoaminergic neuromodulation of circuits intrinsic to the prefrontal cortex, sensorimotor cortex, and the basal ganglia.^1, 2, 3, 4, 5^ With continued, long-term treatment, symptoms continue to gradually improve.^6^ However, the underlying neurobiological correlates of this therapeutic effect remain poorly understood.

Studies using anatomical magnetic resonance imaging (MRI) suggest that individuals with ADHD exhibit reduced volumes of various cortical regions,^1, 7, 8^ as well as the basal ganglia, particularly of nuclei comprising the striatum.^2, 9, 10, 11^ The striatum is the main input region of the basal ganglia that plays a role in habit learning, motivation, goal-directed behaviors, reward processing and motor control,^12, 13, 14, 15, 16^ functions that have been shown to be disturbed in ADHD.^17, 18^ Some studies indicate that striatal volume shows evidence of normalization in patients with a history of psychostimulant treatment relative to untreated patients.^19, 20^ Moreover, Sobel *et al.*^9^ found that localized increases in striatal volume in treated patients paralleled symptom improvement. These findings suggest that long-term stimulant treatment may intervene in the disease process and reduce symptom severity by altering striatal morphology (i.e., by stimulating hypertrophy).

Studies investigating striatal morphology in ADHD have in general been limited by the use of cross-sectional designs. The two longitudinal studies that have been performed did not assess the impact of treatment length on dependent outcomes.^7, 21^ These limitations have precluded causal links from being drawn between stimulant treatment and changes in morphology and behavior. Furthermore, little is known about the molecular mechanisms of stimulant-induced changes in striatal morphology. As MRI analyses in humans are limited to detecting macroscopic changes in the brain, animal models are needed to determine the underlying cellular and molecular nature of stimulant-induced alterations in striatal morphology. Ultimately, understanding how these changes relate to performance on behavioral tasks probing impulsivity, attention, and other cognitive domains affected in ADHD,^22^ will provide a crucial step towards developing novel, more targeted treatments with fewer side effects than currently available medications.^23^

The primary aim of the current investigation was to establish causality regarding the effects of long-term stimulant treatment on striatal morphology in a rodent model using comparable MRI technologies to those used in human studies. To test the hypothesis that long-term stimulant treatment induces striatal hypertrophy, we used a vehicle-controlled, longitudinal study design with a rodent model of clinically relevant stimulant administration. In order to track changes in regional volume as a function of treatment and length of exposure, we made novel use of longitudinal *in vivo* rodent MRI techniques to allow for repeated brain measurements in each animal across treatment course (i.e., MRI scanning before and after treatment in each group). In addition, we set out to confirm our study findings by calculating regional volumes, by an independent rater, in *ex vivo* gadolinium-contrasted high-resolution MRI scans acquired on the same animals that completed the longitudinal study. Given the prominent use of MRI as a research tool in clinical populations, the use of this technology in rodents confers high translational value as findings could be readily compared with clinical samples.

## Materials and methods

### Animals and husbandry

Male, Sprague Dawley rats between the ages of postnatal day (PD) 23–94 were used in the present study. To habituate the animals to oral deposition of drug vehicle before study initiation, we acquired all our experimental animals through timed-pregnant female rats purchased from Charles River Laboratories (Kingston, NY, USA). At birth, males were culled and remained with the mother until weaning (PD21), after which they were pair-housed with siblings receiving the opposite treatment until the end of the study. All rats were housed under a 12 h light/dark cycle in a temperature-controlled environment with food and water available *ad libitum*. The Institutional Animal Care and Use Committee at the Nathan Kline Institute for Psychiatric Research approved of all the animal protocols used in this study.

### Treatment administration

All animals were weighed daily from PD15 until study completion, with treatment adjusted to account for increases in body weight over the course of development. Before the first MRI scan at PD23 (see below), all animals were habituated to the oral delivery of drug vehicle from PD15 until PD22. The vehicle consisted of apple juice (5 ml kg^−1^), which was slowly deposited *per os* using a 1000 μl pipette inserted towards the back of the mouth. One day following the first MRI scan, the animals were treated with either stimulant (*N*=12) or vehicle (*N*=12) for 10 weeks (PD24–94), encompassing the young juvenile period until early adulthood.^24^ Animals were pseudo-randomly assigned to either treatment group, and the sample size was determined on the basis of a prior publication where stimulant-induced increases in striatal volume in adult rats were detected via post-mortem MRI.^25^ The stimulant group received twice daily (morning and afternoon, 5 h apart) 5 mg ml^−1^ kg^−1^ lisdexamfetamine (LDX, ‘Vyvanse'), a prodrug which is metabolized to dextroamphetamine (d-AMPH) once in circulation.^26^ The control group received the same dosing regimen of 1 ml kg^−1^ vehicle only. The 10-week duration of treatment was chosen on the basis of results from the MTA (Multimodal Treatment of ADHD) study where methylphenidate, another stimulant, was found to have maximal effects on symptom improvement in the first 10–12 weeks of the 14-month treatment trial.^6^ On the basis of prior pharmacokinetic assessments of oral LDX in rodents,^27^ the dosing regimen was chosen to approximate peak circulating levels of d-AMPH in humans given a mid-range, 50 mg dose of LDX (93.3±19.5 ng ml^−1^ at the 3 h *T*_max_)^28^ and to mirror the pharmacokinetics of long-release LDX formulations in humans by accommodating for the faster elimination rate of this drug in rodents. Pharmaceutical grade LDX dimesylate was kindly provided by Shire US (Wayne, PA, USA) and solutions were freshly prepared on a daily basis.

### Analysis of peak plasma levels of dextroamphetamine

During the dosing period, a subset of rats in both the LDX and vehicle group (*n*=5 per group) underwent blood extraction at PD30 to measure plasma levels of d-AMPH following the first daily dose of either LDX or vehicle. Blood was extracted at the *T*_max_ for LDX-derived d-AMPH (3 h in Sprague Dawley rats^27^) through the retro-orbital plexus by heparinized capillary tubing following anesthesia with 50 mg kg^−1^ ketamine (intramuscular) under isofluorane (3% isoflurane in 75% NO_2_ and 25% O_2_). Samples were centrifuged at 1000 *g* to recover plasma, which was then mixed 1:10 in phosphate-buffered saline (150 mm NaCl, 0.1 m Na_2_HPO_4_/NaH_2_PO_4_; pH 7.4) before analysis using gas chromatographic-mass spectrometric (GC/MS) instrumentation with deuterated d-AMPH as the internal standard. The system was operated in the negative chemical ionization (NCI) mode with 5% ammonia/methane as the reactant gas. After protein precipitation of 0.5 ml of each plasma sample, the supernatant was made alkaline, derivitized with pentafluorobenzoyl chloride, and simultaneously extracted into toluene. An aliquot of the toluene extract was then injected into the GC/MS equipped with a DB-1 15 m × 0.25 mm ID, 0.25 μm film capillary column. The generated standard curve was linear with a limit of detection of 1 ng ml^−1^. Intra- and inter-assay precision was better than 94 and 98%, respectively.

### Longitudinal *in vivo* MRI

All study animals had two anatomical MRI scans: first, one day before treatment initiation (PD23), and second, one day following treatment completion (PD95). Scanning was performed on a 7.0 Tesla Agilent (Santa Clara, CA, USA) 40 cm bore system. The gradient coil insert had an internal diameter of 12 cm, a maximum gradient strength of 600 mT m^−1^, minimum rise time of 200 μs, and customized second- and third-order shim coils. A Rapid (Rimpar, Germany) volume transmit coil (72 mm ID) and a two-channel (PD23) or a four-channel (PD95) receive-only surface coil were used for radiofrequency (RF) transmission and reception. A magnetization prepared rapid acquisition gradient echo sequence was used for image acquisition.^29^ The isotropic image resolution for PD23 animals was 0.175 × 0.175 × 0.175 mm^3^, and for PD95 animals was 0.2 × 0.2 × 0.3 mm^3^. The inversion time was 1.1 s, the segment repetition time 3.4 s, and total acquisition time 1 h using four averages. To prevent head motion and anxiety during the scan, all animals were anesthetized with isofluorane gas. Oxygen was used as the carrier gas and delivered at low flow rate (⩽0.5 l min^−1^) to a cone positioned before the bite bar. Before each scan, animals were exposed to 3% isofluorane for induction and then secured in the RF coil with an integrated bite bar, stereotaxic holder, and anesthesia mask. During the scans, isofluorane anesthesia was reduced to 1.5%. Monitoring of the heart rate, respiratory rate and O_2_ was performed using an SA Instruments monitoring unit (Model 1025, Stony Brook, NY, USA) during the whole procedure. Body temperature was monitored through a rectal probe, and was maintained at 37±0.2 °C by a forced warm air unit interfaced to the SA Instruments unit.

### *Exvivo* high-resolution MRI

At the conclusion of the final scan, all animals were removed from isofluorane and deeply anesthetized with a ketamine/xylazine mixture (100 mg kg^−1^, 20 mg kg^−1^) delivered by intraperitoneal injection. Animals were then euthanized through transcardial perfusion with 4% paraformaldehyde in phosphate buffer (0.1 m Na_2_HPO_4_/NaH_2_PO_4_; pH 7.4), after which the head was removed and processed for gadolinium-contrasted *ex vivo* high-resolution MRI. Briefly, the whole head was post-fixed in 4% paraformaldehyde buffer (pH 7.4) on a slow-rocking shaker overnight at 4 °C. The head was then stripped to the skull and transferred to 0.05% sodium azide in phosphate buffer for 1 week at 4 °C. The skull was then incubated in a solution of 0.3% gadolinium (Multihance, Bracco Diagnostics, Monroe Township, NJ, USA) in 0.05% sodium azide/phosphate buffer and gently rocked for 2 weeks at 4 °C. For imaging, the skull was placed in a solution of Fomblin Y (Sigma-Aldrich, St.Louis, MO, USA), which has no MR signal, to eliminate any background saturation artifacts. For each sample, a high-resolution anatomical MRI scan was acquired for 9 h and 49 min overnight, using the volume transmit coil, the four-channel receive surface coil and Spin Echo three-dimensional acquisition with 80 μm isotropic resolution (repetition time=120 ms, echo time=18 ms). Following the scan, the brains were removed from the skull, repeatedly washed in phosphate buffer to remove residual Fomblin Y, and weighed for subsequent normalization procedures (see the subsection 'Regional Volume Calculation and Normalization', below).

### Manual tracing of MRI images

Manual delineation of regions of interest was performed using itk-SNAP software (http://www.itksnap.org) on the *in vivo* (i.e., PD23 and PD95) as well as high-resolution *ex vivo* (PD95) MRI scans, each modality by an independent rater. Landmarks for tracing were established according to the segmented Sprague Dawley brain atlas (http://rbwb.org, Papp *et al.* ^30^). The whole striatum was traced along an axial plane in each hemisphere in all scans. racing of the thalamus and hippocampus was also performed but only on *ex vivo* MRI scans, as lower resolution and signal-to-noise ratio of the longitudinal *in vivo* scans prevented clear delineation of these structures (see example images from each modality in Figure 2).

### Regional volume calculation and normalization

Structural volume (mm^3^) of traced regions was calculated using standard itk-SNAP algorithms. For analyses of the *ex vivo* high-resolution MRI, regional volumes were normalized by individual brain weight. This normalization procedure was performed to appropriately scale volumes of individual structures to overall brain size, which was reduced in the LDX-treated animals (see the 'Results' section, Supplementary Figure 1). For secondary analyses of the *in vivo* longitudinal MRI, due to the longitudinal design of our study, we could not obtain brain weight measures for the PD23 animals; instead, regional volumes were normalized by individual body weight at each time point, which was also reduced in the LDX-treated animals (see the 'Results' section, Figure 2). Supporting the use of body weight as a proxy measure of brain weight for these analyses in our study, (i) we found a strong, significant correlation between body weight and brain weight in our PD95 animals (see the 'Results' section, Supplementary Figure 2) and (ii) it has previously been shown that body weight across age in rodents is proportional to brain volume as measured by anatomical MRI.^31^ The normalized volumes across groups at each time point were then transformed to *t*-scores to allow standardized computation of differences between groups with repeated-measures analysis.

### Exploratory analysis

For analysis of striatal subregions in the *ex vivo* high-resolution MRI, we first used itk-SNAP to export three-dimensional masks of each individually traced striatal structure from each animal. As tracing was performed along an axial plane, we used FSL to segment these masks into three equal parts (based on the number of traced layers) comprising the approximate dorsal, mid, and ventral aspects of the striatum (see Figure 2h for illustration). FSL was then used to compute the volume of each segment, which was normalized to each respective animal's brain weight for subsequent group comparisons.

### Statistical analysis

SPSS 21 (Armonk, NY, USA) was used for all statistical comparisons. A *P*⩽0.05 was considered statistically significant. All the data are presented as mean±s.e.m.

### Body and brain weight

Animal weight across the treatment period (PD24–94) was compared between groups by repeated-measures analysis of variance (ANOVA) with treatment as the between-subject factor, and age as the within-subject factor. Significant interactions were examined with *post hoc t*-tests. At the completion of the study (i.e., PD95), brain weight between the treatment groups was compared with a *t*-test. Pearson's correlation was used to assess the relationship between brain and body weight at PD95.

### Main hypothesis testing: longitudinal *in vivo* MRI

For analysis of longitudinal *in vivo* MRI (i.e., at PD23 and PD95), striatal volumes (mm^3^) were compared between treatment groups with a 2 × 2 repeated-measures ANOVA with treatment as the between-subject factor, and time point as the within-subject factor. Significant interactions were examined with *post hoc t*-tests. Analyses were first conducted on raw striatal volumes and then confirmed with normalized striatal volumes.

### Confirmatory analysis: high-resolution *ex vivo* MRI

For the *ex vivo* MRI (PD95), normalized volumes within three regions of interest (striatum, thalamus and hippocampus) in LDX- vs vehicle-treated animals were compared cross-sectionally with a *t*-test.

### Exploratory analysis: high-resolution *ex vivo* MRI

Volumetric differences between striatal subregions (i.e., dorsal, mid or ventral) across both hemispheres were assessed by repeated-measures ANOVA with treatment as the independent variable, and subregions/hemisphere as the dependent variables. To estimate the magnitude of treatment effects on subregional volumes, we computed the effect size (partial eta-squared) of each between-group *t*-test comparison of each subregion/hemisphere combination.

## Results

### Peak plasma levels of lisdexamfetamine-derived dextroamphetamine

On the basis of a blood sample draw at the 3 h *T*_max_ (ref. 27) from a subset of PD30 animals, we found that our LDX treatment regimen yielded peak plasma levels of d-AMPH of 96.8±17.04 ng ml^−1^. This is comparable to peak circulating levels of d-AMPH in children with ADHD given a mid-range dose of 50 mg LDX (93.3±19.5 ng ml^−1^ at *T*_max_),^28^ indicating that our dosing regimen of oral LDX in rats yielded clinically relevant levels of plasma d-AMPH. d-AMPH was not detected in the plasma of vehicle-treated animals.

### Body weight across treatment

**Figure 1:**
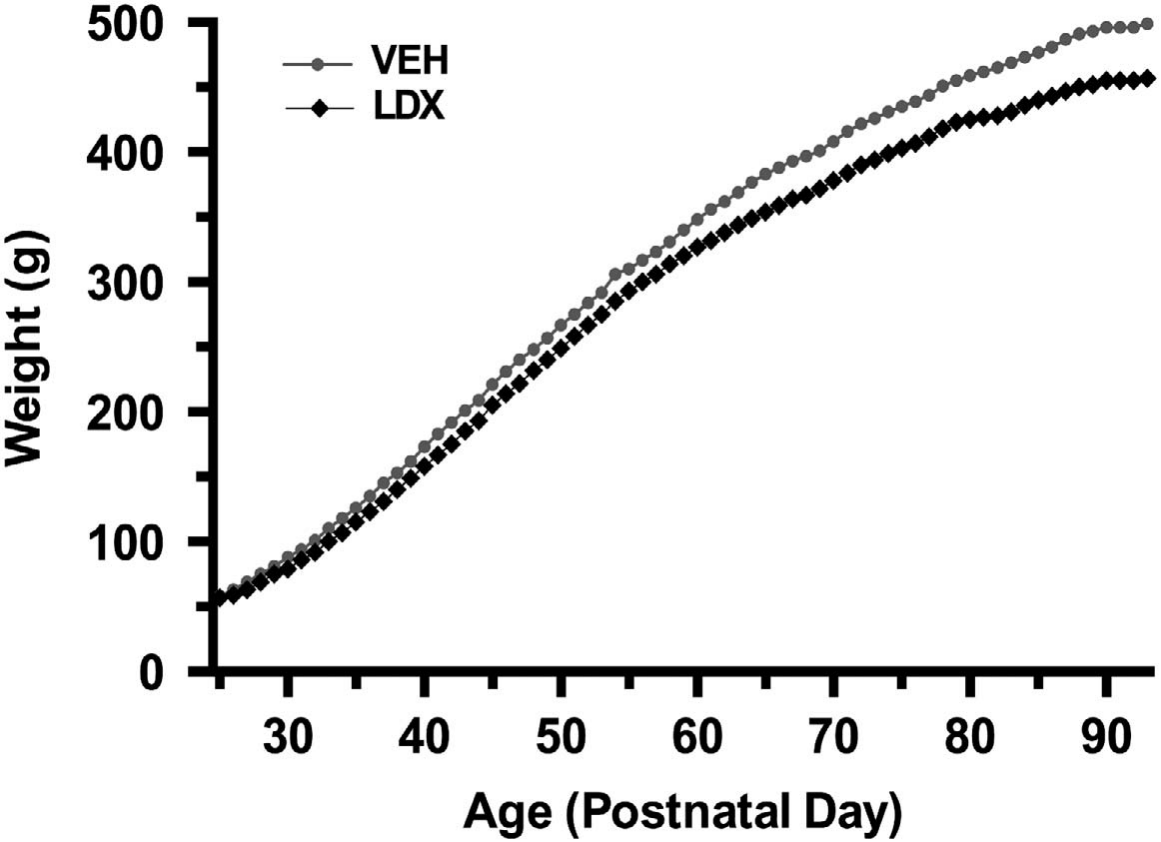
Body weight across treatment. Relative to vehicle-treated animals (VEH, *N*=12), animals administered lisdexamfetamine (LDX, *N*=12) gained significantly less weight across the 70-day dosing period from postnatal day 24 (PD24) to PD94, with a significant treatment by age interaction.

As revealed by repeated-measures ANOVA, body weight showed a significant treatment × time point interaction (*f*(68,1496)=5.315, *P<*0.0001), with LDX-treated animals weighing significantly less than vehicle-treated controls by the end of treatment (PD94: 452.50±13.89 g vs 498.92±11.70 g, *t*(22)=−2.56, *P*=0.02; Figure 1). Body weight between LDX- and vehicle-treated animals did not differ at the start of the dosing period (PD24: 57.00±1.81 g vs 58.00±1.78 g, *t*(22)=−0.39, *P*=0.70). These results are consistent with the known appetite-suppressant effects of stimulant treatment in human patients.^32^

### Brain weight following treatment

At the completion of the study, PD95 animals in the LDX group showed a significant reduction in brain weight when compared with vehicle-treated controls (2.20±0.06 g vs 2.52±0.06 g, *t*(22)=−3.49, *P*=0.002; Supplementary Figure 1).

### Brain weight vs body weight correlation

Across all PD95 animals (both the LDX and vehicle group), brain weight was found to be significantly correlated with body weight (*r*(24)=0.519, *P*=0.005; Supplementary Figure 2).

### Main hypothesis testing

#### *Longitudinal* in vivo *MRI: raw striatal volumes*

**Figure 2:**
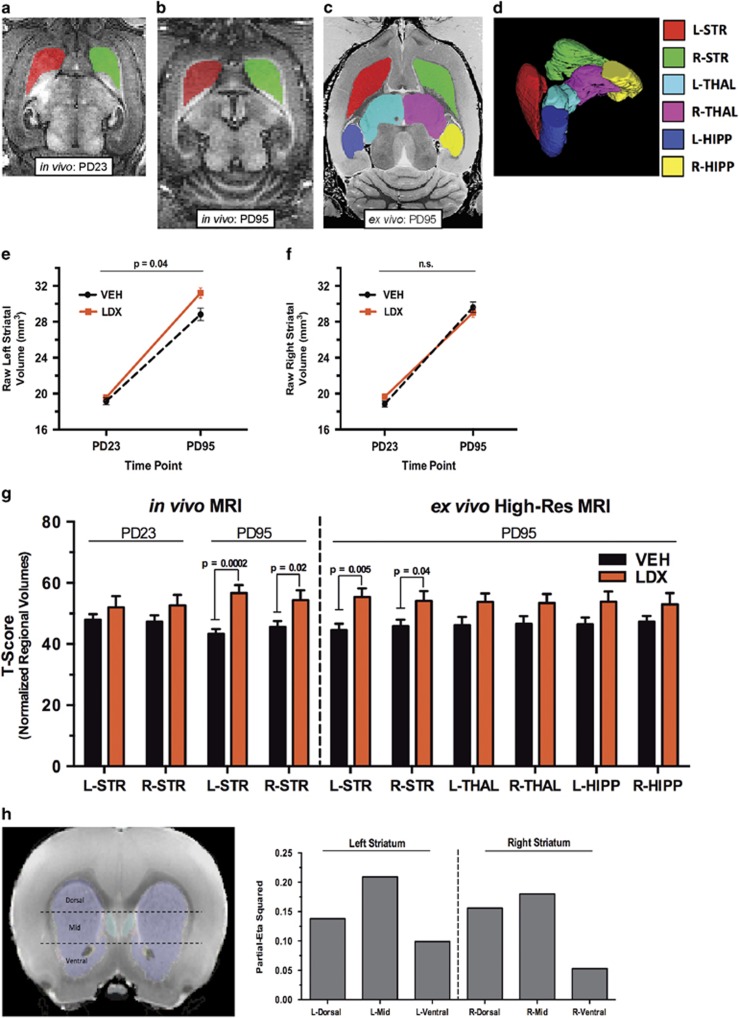
Region of interest volumes computed from longitudinal *in vivo* MRI and high-resolution *ex vivo* MRI. (**a**, **b**) Representative tracings of the left (L) and right (R) striatum (STR) in repeated, longitudinal *in vivo* anatomical MRI scans acquired from the same animal at postnatal day 23 (PD23; **a**) and PD95 (**b**). (**c**) Representative tracings of the left and right STR, thalamus (THAL) and hippocampus (HIPP) in an *ex vivo* high-resolution (High-Res) anatomical magnetic resonance imaging (MRI) scan acquired from the same PD95 animal as in **a** and **b**. (**d**) The color legend for **a**–**c** is shown here, including an example three-dimensional (3D) reconstruction of each traced region computed from the High-Res scan as in **c**. (**e**, **f**) Graphs depict differences in raw volumes of the left (**e**) and right (**f**) striatum between animals treated for 70 days (PD24–94) with either lisdexamfetamine (LDX) or vehicle (VEH; *N*=12 per group). No statistical differences were found between the groups at PD23. At PD95, relative to VEH-treated controls, the animals treated with LDX showed significant hypertrophy of the left but not right striatum, with left striatal volume showing a significant treatment by time point interaction (*P*=0.04). (**g**) Graph depicts *t*-score differences in normalized regional volumes between treatment groups computed from longitudinal *in vivo* MRI (left) and cross-sectional *ex vivo* High-Res MRI (right). Longitudinal *in vivo* MRI: no statistical differences in striatal volume were found at PD23. At PD95, relative to VEH-treated controls, animals treated with LDX showed significant hypertrophy of the left (*P*=0.0002) and right (*P*=0.02) striatum. Only the left striatum showed a significant treatment by time point interaction. High-Resolution *ex vivo* MRI: relative to VEH-treated controls, the animals treated with LDX showed significant hypertrophy of the left (*P*=0.005) and right (*P*=0.04) striatum. Neither the thalamus nor the hippocampus showed a significant group difference in volume. (**h**) Exploratory analysis of LDX effects on subregional striatal volume at PD95 (*ex vivo* High-Res MRI). The illustration to the left depicts the approximate coronal location of segments comprising the dorsal, mid and ventral striatum as derived from FSL-based segmentation of subject-specific tracing masks (see the 'Materials and methods' section). Partial eta-squared effect sizes were computed from between-group *t*-test comparisons of normalized volume of each subregion within each hemisphere. As shown in the graph on the right, these analyses revealed that within both hemispheres (i.e., left and right striatum), LDX treatment yielded the largest effect on striatal hypertrophy in the mid and dorsal striatum, with a relatively smaller effect on the ventral aspect of this structure. MRI, magnetic resonance imaging.

A 2 × 2 repeated-measures ANOVA of left striatal volume revealed a significant treatment × time point interaction (*f*(1,22)=4.53, *P*=0.04; Figure 2e). This interaction was not significant for the right striatum (*f*(1,22)=2.90, *P*=0.10; Figure 2f). *Post hoc* testing revealed no significant difference between the groups in left or right striatal volume at PD23 (left striatum: *t*(22)=0.81, *P*=0.43; right striatum: *t*(22)=1.70, *P*=0.10). At PD95, relative to controls, the LDX group showed a significant increase in volume of the left (*t*(22)=2.68, *P*=0.0014) but not right striatum (*t*(22)=−0.75, *P*=0.461; Figure 2e and f).

#### *Longitudinal* in vivo *MRI: normalized striatal volumes*

All comparisons are shown in Figure 2g. A 2 × 2 repeated-measures ANOVA of left striatal volume revealed a significant treatment × time point interaction (*f*(1,21)=4.89, *P*=0.04). This interaction was not significant for the right striatum (*f*(1,21)=0.62, *P*=0.42). *Post hoc* testing revealed no significant difference between the groups in left or right striatal volume at PD23 (left striatum: *t*(22)=1.00, *P*=0.16; right striatum: *t*(22)=1.34, *P*=0.10). At PD95, the LDX group showed a significant increase in volume of the left and right striatum, relative to controls (left striatum: *t*(22)=4.43, *P*=0.0002; right striatum: *t*(22)=2.34, *P*=0.02).

### Confirmatory analysis

#### *High-resolution* ex vivo *MRI: normalized region of interest volumes*

All comparisons are shown in Figure 2g. *T*-tests revealed significantly higher volume of the left striatum (*t*(22)=3.11, *P*=0.005) and right striatum (*t*(22)=2.166, *P*=0.04) in LDX-treated animals relative to vehicle-treated controls. Neither the thalamus nor the hippocampus showed a significant group difference in volume (left thalamus: *t*(22)=1.95, *P*=0.06; right thalamus: *t*(22)=1.76, *P*=0.09; left hippocampus: *t*(22)=1.87, *P*=0.08; right hippocampus: t(22)=1.39, *P*=0.18).

### Exploratory analysis

#### *High-resolution* ex vivo *MRI: normalized subregional volumes*

The repeated-measures ANOVA did not show a statistically significant between-group difference in volumes of striatal subregions (i.e., dorsal, mid or ventral) across the left and right hemisphere (*f*(1,22)=3.842, *P*=0.063). To explore trends in our data, we performed between-group *t*-test comparisons of each subregion within each hemisphere. This exploratory analysis revealed the largest effect size of LDX treatment on volume in the mid (partial eta-squared: left=0.209; right=0.180) and dorsal (partial eta-squared: left=0.138; right=0.156) striatum, with a relatively smaller effect in the ventral aspect of this structure (partial eta-squared: left=0.099; right=0.053). This pattern of effects was consistent between both hemispheres (Figure 2h).

## Discussion

We used longitudinal MRI in rodents in the context of a prospective, vehicle-controlled design to test the hypothesis that long-term treatment with clinically relevant doses of the stimulant LDX produces striatal hypertrophy. In agreement with this hypothesis, we found that relative to vehicle-treated controls, treatment of rats with LDX between juvenility (PD25, first scan) and young adulthood (PD95, second scan) induced significantly greater striatal growth, with the left striatum showing a stronger effect and a significant treatment by time point interaction. To test the reliability and specificity of these findings, region of interest analyses were performed by an independent rater on high-resolution *ex vivo* MRI scans acquired from the same PD95 animals as were used in the main, longitudinal study. These analyses confirmed that, relative to controls, the striatum shows significantly greater volume in LDX-treated animals. In addition, exploratory analyses, albeit at a trend level, suggested that the effect of LDX on striatal hypertrophy may have been greater in mid-dorsal, relative to ventral, subregions of the striatum. These volumetric effects were specific to the striatum, as volumes of the thalamus and hippocampus did not statistically differ between treatment groups as measured in the high-resolution scans.

The striatum is a major input nucleus of the basal ganglia that is implicated in the pathophysiology of ADHD and other conditions such as addiction, schizophrenia and obsessive-compulsive disorder.^33, 34, 35^ Many of the symptoms associated with ADHD respond to acute treatment with stimulants and symptom improvement is often noted within 30–60 min of oral drug administration. With maintenance of treatment, continued symptom reductions are reported over the subsequent 10–14 months.^6^ Although the acute phase of these therapeutic effects is thought to result from altered monoaminergic modulation of circuits underlying attention, impulsivity, and motivation,^36^ continued improvement in symptoms as a function of prolonged treatment is not well understood. Using neuroanatomical imaging of individuals with ADHD, studies have found that in comparison to youth with ADHD who are medication-naive, those with a history of stimulant therapy exhibit larger striatal volume,^19, 20^ with morphometric analyses finding localized increases in volume that correlate with symptom improvement.^9^ Although limited by their cross-sectional design, these studies suggest that striatal hypertrophy might be a neurobiological correlate of continued improvement in ADHD symptoms stemming from long-term stimulant treatment. Other studies, however, have not reported stimulant-induced effects on striatal volumes in ADHD.^10, 37, 38^ These discrepancies in findings could potentially relate to methodological issues such as the developmental stage (childhood vs adolescence), stimulant dosing or class (methylphenidate vs amphetamines), duration of treatment, sample size, or technical approach for assessing striatal morphology.^20^ Moreover, it should be noted that two longitudinal studies have been performed to investigate the trajectory of volumetric changes in basal ganglia structures between medicated and unmedicated patients with ADHD and healthy controls. These studies have been discordant, with Castellanos *et al.*^7^ finding that initial differences in striatal volume between patients with ADHD and healthy controls disappear by adolescence, while Shaw *et al.*^21^ showing that these initial differences persist into adolescence/adulthood. In addition, neither study found an association between basal ganglia volume and treatment history. However, both studies treated medication status as a dichotomous variable and thus did not assess the impact of the dose or duration of exposure on striatal morphology. Future longitudinal studies emphasizing prospective, chronic treatment with stimulants are therefore needed to confirm these effects and their relationship to symptom prognosis.

Despite continued improvement in ADHD symptoms with chronic stimulant treatment (for example, 14-month duration),^6^ the majority of stimulants' salutary effects occur through acute drug action. It is unlikely that volumetric changes in the striatum, which may occur gradually as a function of stimulant treatment, account for the immediate benefits of stimulant treatment on symptom improvement. However, structural changes in the striatum in medicated individuals with ADHD have been associated with improvement in clinical outcomes.^9^ Taken together, a potential implication of our findings is that increases in striatal volume across development, stemming from either normative neurodevelopment, or its interaction with long-term stimulant treatment, may impact the natural progression of ADHD in affected individuals. For example, over 50% of children with ADHD will remit by early adulthood.^39^ Future studies could test the hypothesis that striatal maturation, at least in part, accounts for this attenuation of ADHD symptoms, and whether long-term treatment with stimulants augments this progression by arcing striatal growth towards a normative neurodevelopmental trajectory. In addition, exploratory analyses suggested that the effects of LDX on striatal hypertrophy might be more pronounced in the mid/dorsal, than ventral, subregions of this structure. Subsequent research might investigate in both humans and preclinical models the behavioral correlates of pharmacologically induced hypertrophy of dorsal vs ventral striatal subregions.

In addition to measuring striatal volume in our study, we assessed the effects of long-term stimulant treatment on thalamic and hippocampal volume. As the thalamus receives indirect input from the striatum through well-established cortico–striatal–thalamic–cortical loops,^40, 41^ analysis of this region tested the specificity of stimulant effects on the striatum vs cortico–striatal–thalamic–cortical circuitry in general. The hippocampus was included as a negative control as stimulant treatment has been shown to cause nonsignificant volumetric changes in this structure in rodents.^25, 42^ As we did not detect a volumetric change in either the thalamus or hippocampus in our study, our findings suggest that the effects of long-term stimulant treatment may be specific to the striatum and not cortico–striatal–thalamic–cortical loops or other brain regions more broadly. In addition, the hippocampal finding verified that our analytical procedures, such as adjusting for brain weight, did not yield biased estimates of striatal volume between groups. It has yet to be determined, however, whether stimulant-induced striatal hypertrophy impacts striatal activity and cortico–striatal–thalamic–cortical function, and what consequences this may have for behavior.

A general limitation of this study is that it does not use an animal model of ADHD. This is important to consider since stimulant effects on the brain may differ in children with ADHD when compared with healthy individuals. Although animal models of ADHD do exist (for example, spontaneously hypertensive rats),^43^ ADHD is a highly heterogeneous disease with presumably many different etiologies and pathophysiologies.^44, 45^ Thus the use of such models may limit the extrapolation of findings to only a small subset of ADHD cases.^46^ However, despite the heterogeneity of ADHD, up to three-quarters of patients respond well to stimulant treatment,^47, 48^ implying that generalizable effects on the brain may underpin the therapeutic response to stimulants irrespective of individual pathology, thus supporting our use of a ‘typical' rat model of stimulant exposure. In addition, our tracing procedure did not allow us to discern the impact of stimulant treatment on functionally distinct areas of the striatum, such as those comprising the associative, limbic, and motor circuits.^49, 50^ Future studies may address this issue by performing surface analysis of the striatal structure, which can detect localized changes in morphometry. The use of post-mortem high-resolution MRI in our study did not allow us to determine the nature of the volumetric changes. Although studies have shown that chronic stimulant exposure can lead to increased dendritic arborization and spine formation,^51^ an increase in volume may also result from alterations in cellular number, somatic size, myelination, or innervation.^52^ As MRI analysis is limited to detecting macroscopic changes in the brain, future studies will be needed to elucidate the cellular and molecular underpinnings of stimulant-induced striatal hypertrophy. A number of studies have suggested that delayed maturation of the cortex may play a major role in ADHD pathology,^7, 53^ although evidence of normalization with stimulant treatment has been controversial.^21, 53, 54^ Without MRI scans at least one additional developmental period (for example, adolescence), our study design precluded us from assessing the effects of long-term stimulant treatment on nonlinear growth trajectories of cortical regions.^55^ Modeling nonlinear patterns of brain development requires assessments from at least three time points.^56, 57^ Finally, although our finding of striatal hypertrophy in our rodent model of long-term stimulant treatment recapitulates human findings, we did not assess whether this alteration is associated with behavioral outcomes. Given the possibility that the salutary effects of stimulants on behavior in humans may in part depend on the presence of a pathological state,^58^ future studies may evaluate the clinical application of our rodent-stimulant model by investigating whether stimulant-induced increases in striatal volume yield behavioral changes in symptom domains relevant to ADHD such as impulsivity, attention,^36^ and aggression.^59^

The long-term goal of our study is to invoke a translational neuroscience approach towards identifying the cellular and molecular mechanisms by which clinically relevant doses of stimulants improve the symptoms of ADHD. This study is an important, initial step in that direction. To our knowledge, this is the first demonstrated use of repeated, longitudinal *in vivo* MRI in rodents to measure brain changes as a function of treatment and natural brain development. Use of this technique has high translational potential to serve as a tool for bridging preclinical and clinical research. As our findings of stimulant-induced striatal hypertrophy in rodents agree with what has previously been found in humans, this suggests high translational validity of our rodent model of long-term stimulant exposure. The principal advantage of our animal model is that mechanisms underlying this effect can be simultaneously investigated at a molecular, cellular, and circuit level, which can then be related to alterations in behaviors relevant to those disrupted in ADHD. Such investigations may yield novel, more targeted approaches for ADHD therapy.
