# Supplementary material for: Longitudinal magnetic resonance imaging reveals striatal hypertrophy in a rat model of long-term stimulant treatment
Source: Transl Psychiatry. 2016 Sep 6;6(9):e884–. doi: 10.1038/tp.2016.158 (PMC5048200; doi:10.1038/tp.2016.158)
Supplement: Supplementary Figures [file tp2016158x1.doc]

Supplementary Information

**Longitudinal Magnetic Resonance Imaging Reveals Striatal Hypertrophy in a Rat Model of Long-Term Stimulant Treatment**

**Running Title: Stimulant-Induced Striatal Hypertrophy**

**Dominik Biezonski, PhD^a^,*, Relish Shah, MS^b^, Anastasia Krivko, MD^a^, Jiook Cha, PhD^a^, David N. Guilfoyle, PhD^b^, , Jan Hrabe, PhD^b^, Scott Gerum, MS^b^, Shan Xie PhD^b^, Yunsuo Duan, PhD^a^, Ravi Bansal, PhD^d^, Bennett L. Leventhal, MD^c^, Bradley S. Peterson, MD^d^, Christoph Kellendonk, PhD^a#^, Jonathan Posner, MD^a#^**

^#^Shared Senior Authors

^a^ Department of Psychiatry, Columbia University College of Physicians and Surgeons and New York State Psychiatric Institute, New York, NY 10032, USA

^b^ Center for Biomedical Imaging and Neuromodulation, Nathan S. Kline Institute for Psychiatric Research, Orangeburg, NY 10962, USA

^c^ Langley Porter Psychiatric Institute, University of California San Francisco, San Francisco, CA 94143, USA

^d^ Institute for the Developing Mind, Children’s Hospital Los Angeles and the Keck School of Medicine at the University of Southern California, Los Angeles, CA 90033, USA

**Contents:**

**- Supplementary Figures**

**SUPPLEMENTARY FIGURES**

**
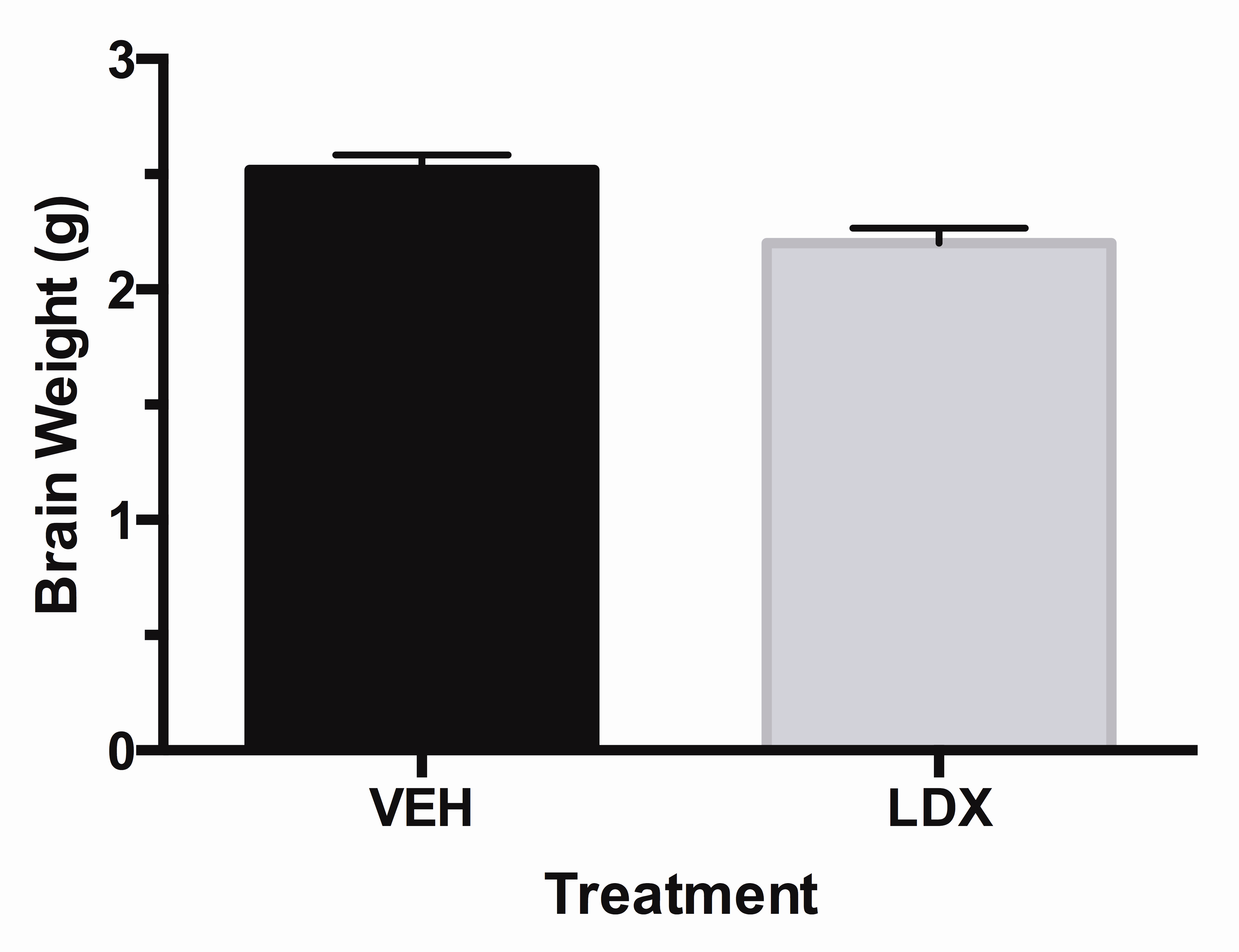
**

**Supplementary Figure 1.** Brain Weight Following Treatment. At the completion of the study, PD95 animals in the LDX group showed a significant reduction in brain weight when compared to vehicle-treated controls (p = 0.002).

**
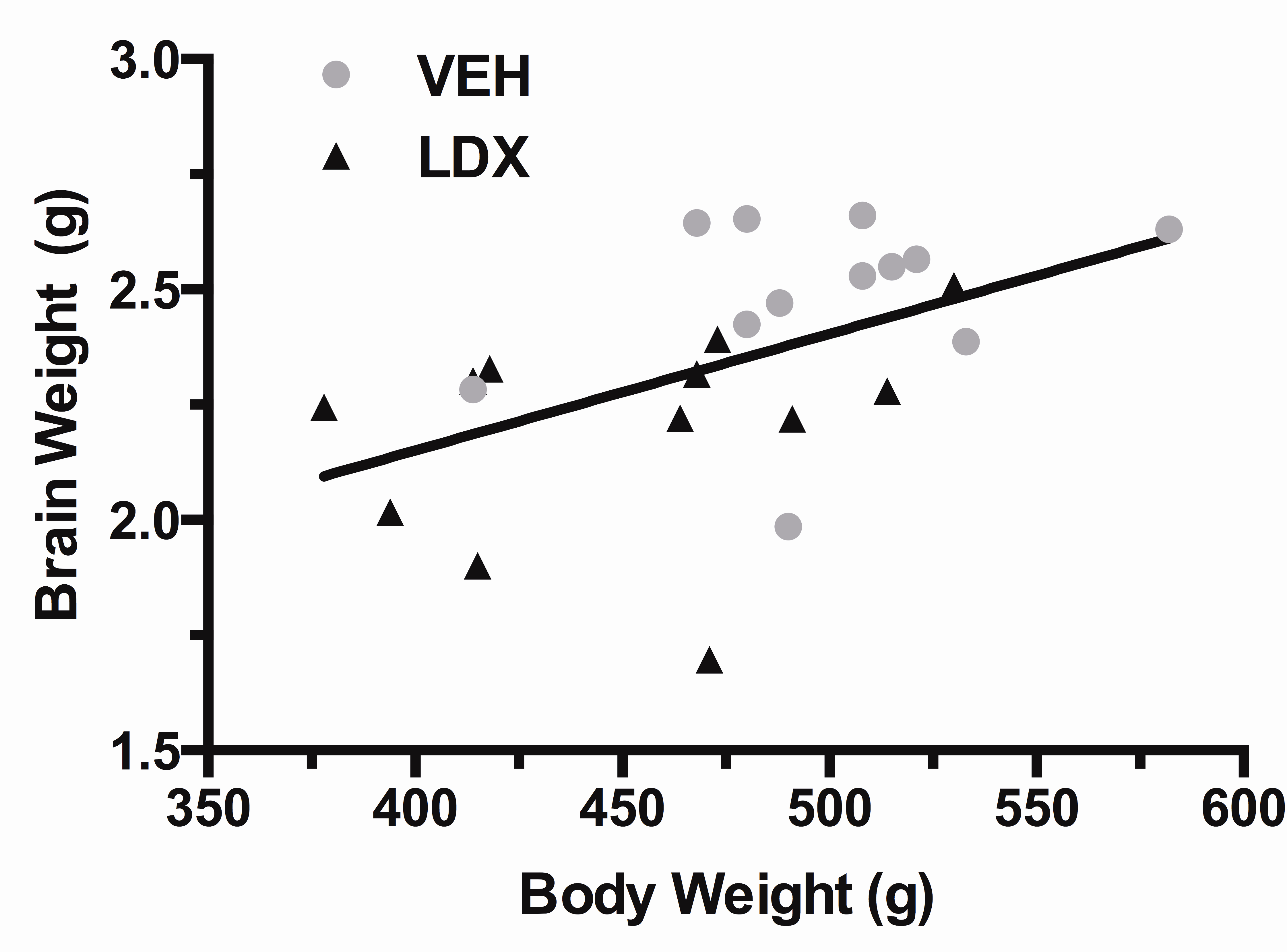
**

**Supplementary Figure 2.** Brain Weight vs. Body Weight Correlation. Across all PD95 animals (both the LDX and VEH group), brain weight was found to be significantly correlated with body weight (r(24) = 0.519, p = 0.005).
